# Vulvar intraepithelial neoplasia grade 3 associated with Behçet’s disease

**DOI:** 10.1186/s13104-016-1985-7

**Published:** 2016-03-17

**Authors:** Daphné Germann, Rosa Catarino, Essia Saiji, Thomas Daikeler, Patrick Petignat, Pierre Vassilakos

**Affiliations:** Department of Gynecology and Obstetrics, Geneva University Hospitals, Geneva, Switzerland; Department of Pathology, Geneva University Hospitals, Geneva, Switzerland; Department of Rheumatology, Basel University Hospitals, Basel, Switzerland; Geneva Foundation for Medical Education and Research, Geneva, Switzerland

**Keywords:** Behçet’s disease, Genital ulceration, Human papillomavirus, Vulvar intraepithelial neoplasia

## Abstract

**Background:**

Little information is available regarding the association between vulvar intraepithelial neoplasia grade 3 (VIN3) and Behçet’s disease (BD). We report here concomitant VIN3 and genital ulcers in a patient with BD.

**Case:**

A 44-year-old Caucasian woman with a history of BD, which had been evolving for 6 years, presented with ulcerated and papillomatous lesions on the vulva. Biopsies revealed a multifocal VIN3 positive for high-risk human papillomavirus (HPV) 33. Multiple biopsies were performed to exclude invasive cancer and VIN3 was treated with laser vaporization.

**Conclusion:**

We report clinical and anatomopathological features of a rare case of multifocal, high-risk, HPV-related VIN3. We also discuss the possible pathogenesis in the context of BD, featuring chronic ulceration and intrinsic or treatment-induced immunosuppression.

## Background

Behçet’s disease (BD) is a rare multisystemic and chronic autoinflammatory disease of unknown origin. Recurrent oral and genital aphthous lesions are nearly always present. Ocular inflammation, and neurological and vascular manifestations are among the most severe complications of BD. Genital ulcerations are usually painful and develop around the anus, vulva, or scrotum, and may be at the origin of scar formation. Coexistence of BD and malignant tumors is rare [1], but association with genital neoplasms has been described in previous reports [2]. To the best of our knowledge, the association between BD and vulvar intraepithelial neoplasia grade 3 (VIN3) has not yet been described in the literature. We report here a woman who was affected by BD, presenting with vulvar ulcers coexisting with multifocal VIN3.

## Case presentation

A 44-year-old Caucasian woman, who had a 6-year history of BD, presented with two different types of vulvar lesions, papillomatous and ulcerated lesions. Her medical gynecological history was compatible with persistence of long-term genital high-risk human papillomavirus (HPV), including excision of a previous large loop of the transformation zone, followed by a hysterectomy because of recurrent cervical intraepithelial neoplasia grade 3, 10 years ago.

During the 6-year period, she had typical manifestations of BD, such as recurrent panuveitis, vitreous body hemorrhage, occlusive vasculitis, and oral aphthous lesions. Laboratory genetic analyses by PCR were negative for human leukocyte antigen (HLA)-B27 and positive for HLA-B51 (PCR-SSO typing using Luminex™, Luminex^®^, Austin, USA).

**Fig. 1:**
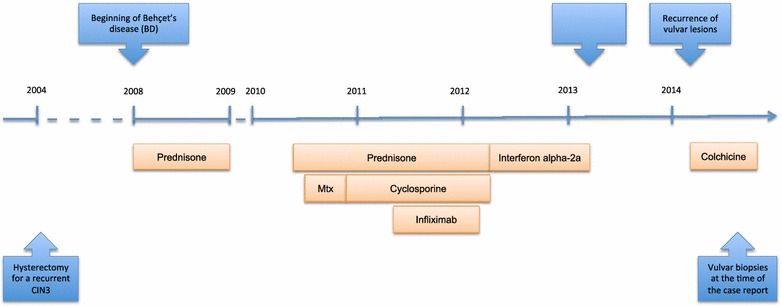
Timeline with main clinical events and patient’s treatment for Behçet’s disease since diagnosis. *Mtx* methotrexate

The patient received various immunosuppressive therapies with prednisone in association with either methotrexate or cyclosporine and infliximab since the initial diagnosis in 2008 (Fig. 1). In 2012, treatment with interferon alpha-2a was introduced and stopped in April 2013 after complete clinical remission of the disease. One year later, she had recurrence, and colchicine was introduced.

**Fig. 2:**
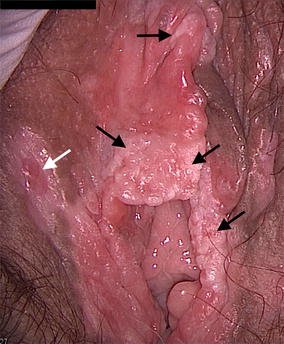
Vulvar clinical examination. Indurated, ulcerated nodule, observed as a benign lesion on biopsy (*white arrow*). White, multifocal, pearly, heightened lesions in the periclitoridian and labia regions can be seen. Multiple biopsies confirmed multifocal VIN3 disease (*black arrows*)

Clinical assessment revealed a highly remodeled vulva with scar formation following recurrent BD (Fig. 2). At the right side, on the labia majora, we observed a slightly indurated and ulcerated nodule, suggestive of a malignant lesion. We also noticed multiple white prominent areas on the labia minora and in the clitoridian region, compatible with a high-grade vulvar neoplasia. Anoscopy under colposcopic vision was normal.

**Fig. 3:**
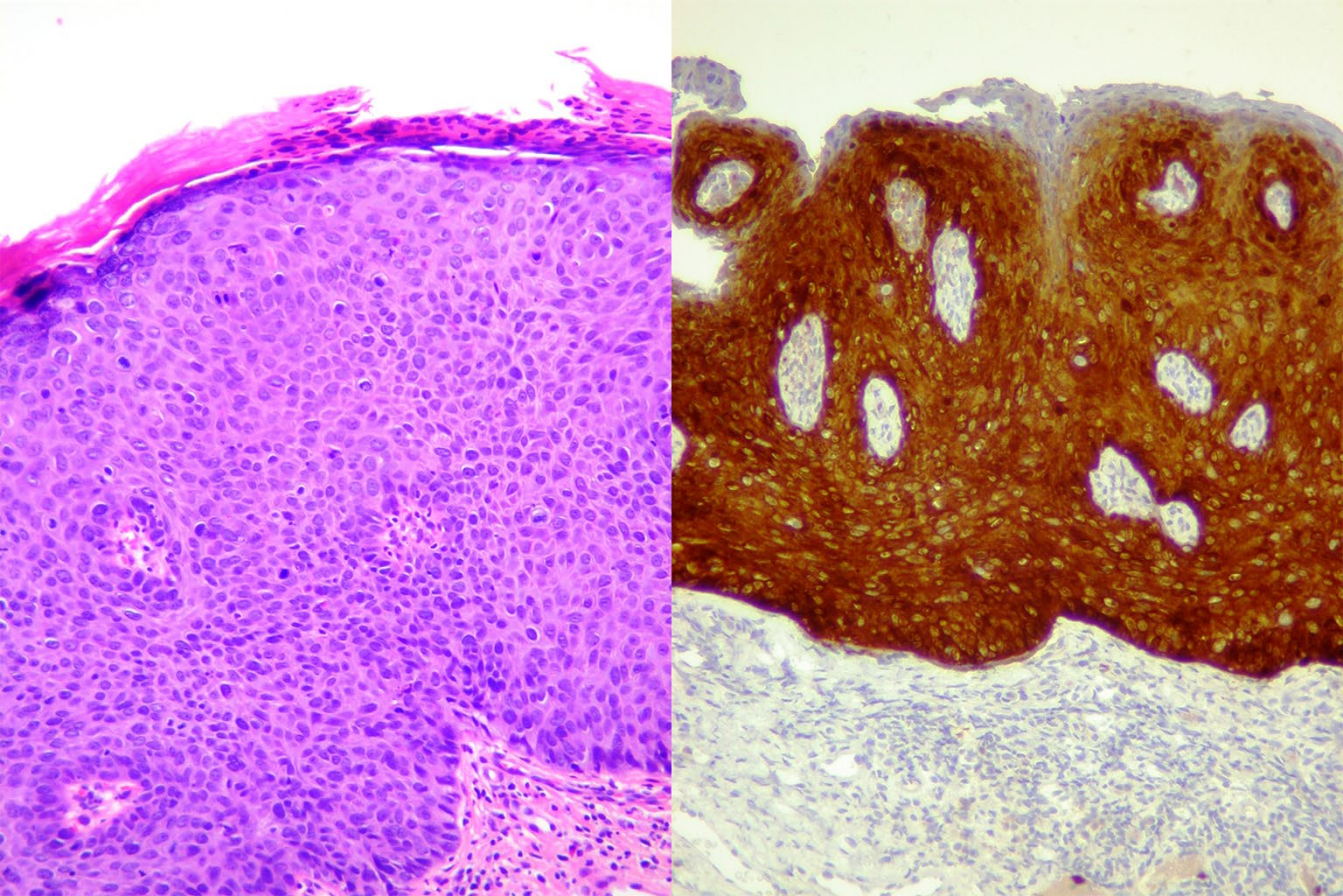
Microscopic findings of vulvar intraepithelial neoplasia grade 3 (VIN3), usual-type VIN. *Left panel* shows usual-type VIN 3, basaloid type, composed of a homogeneous population of abnormal parabasal cells. Hyperchromasia, anisonucleosis, and abnormal mitotic figures in almost the whole thickness of the epidermis can be seen (hematoxylin and eosin, 20×). *Right panel* shows diffuse and strong p16 positivity (p16, 10×)

Under general anesthesia, multiple biopsies were performed to exclude an underlying invasive pathology, and the dysplastic lesions were treated by laser vaporization. A partial right vulvectomy of the labia majora containing an indurated nodule was removed in toto with a margin. Anatomopathological analysis showed an ulcerated Malpighian mucosa. Multifocal biopsies of the labia minora and periclitoridian region confirmed the diagnosis of VIN3 with diffuse and strong p16 immunostaining (Fig. 3). Fresh material from biopsy specimens (VIN3 areas) was analyzed for detection of HPV by a semi-quantitative real-time PCR assay (Anyplex™ II HPV 28, Seegene®, Seoul, Korea) that detects 19 high-risk types and nine low-risk types. The HPV 33 genotype was identified.

The whole intervention was anamnestically well tolerated by the patient.

## Discussion

Although BD has been described as an autoimmune and auto-inflammatory disorder, its etiology still remains unclear [3]. Studies have suggested that BD may be triggered by viral or bacterial antigens. These antigens cause upregulation of interleukins, TNF-α, and interferon-β, leading to activation of neutrophils, which is at the origin of tissue damage. This process is encountered in patients who have a background of genetic susceptibility, such as in the current case, where HLA-B51 was positive. Studies have demonstrated HLA-B51 positivity in more than 60 % of BD patients [4]. This gene is considered as a part of the major histocompatibility complex region of chromosome 6 and is regarded as the primary association with BD within the major histocompatibility complex region [5, 6].

The usual type of VIN is related to high-risk HPV infection and is considered to be the precursor of high-risk, HPV-related, vulvar squamous cell carcinoma. These tumors show multifocality, and occur in younger women, especially if immunocompromised [7]. Pathogenesis of VIN3 in BD patients has not been clearly reported in the literature, but we assume that chronic vulvar inflammation and ulceration may facilitate access of HPV into the basal layer of cells. As infected basal cells mature to the surface, viral replication and viral particle formation and release occurs, spreading the infection into adjacent tissues.

The autoimmune nature of BD and/or immunosuppressive treatment may be considered as a promoter of HPV persistence and evolution toward VIN3, and potentially to cancer. BD-induced immunosuppression might lead to an inability to control the expression of HPV. Production of the HPV oncoproteins E6 and E7 leads to development of precancer and cancer in chronic high-risk HPV infection. In our case, VIN3 was positive for the high-risk HPV 33 genotype and immunostaining showed clear overexpression of the protein p16INK4a. This overexpression, which is present in almost all cervical invasive cancer and precancer, has been shown to be directly linked to the transforming activity of E7 oncoprotein produced by HPV [8].

No data are available regarding the prevalence of HPV in BD and the risk of developing HPV-related, high-grade, intraepithelial neoplasia in the anogenital area. Our patient had multiple genital scars that had formed in an indurated tender area, which can potentially be invasive cancer. Distinction between scars from BD and vulvar cancer may be a challenge in these patients. Our report should alert clinicians about the possibility of high-grade intraepithelial neoplasia in the anogenital area and the possibility of cancer occurring simultaneously with vulvar ulcers. Recognition of this condition is important both clinically and pathologically because small foci of VIN3 may be overlooked when evaluating vulvar lesions associated with BD. Therefore we suggest an accurate clinical gynecological exam with a biopsy for any suspicious area in women who are suffering from BD. Treatment is indicated for VIN3 and after resolution these women should probably be considered as at risk of recurrent VIN and vulvar cancer throughout their lifetimes.

## Conclusions

We report the first known case of high-risk HPV-related VIN3 occurring in association with BD. This case underlines BD as a chronic inflammatory disease of the anogenital area, predisposing to high-grade intraepithelial neoplasia. Prospective studies are needed to evaluate the prevalence of HPV in women with BD and the risk of acquiring anogenital intraepithelial neoplasia. We also recommend careful long-term follow-up of these patients with HPV testing, and biopsy for prompt diagnosis and proper treatment.

## Consent

Written informed consent was obtained from the patient for publication of this case report and any accompanying images.

## Abbreviations

BD: Behçet’s disease
HLA: human leukocyte antigen
HPV: human papillomavirus
VIN: vulvar intraepithelial neoplasia
